# Development and validation of the Accommodation and Enabling Scale for Eating Disorders (AESED) for caregivers in eating disorders

**DOI:** 10.1186/1472-6963-9-171

**Published:** 2009-09-23

**Authors:** Ana R Sepulveda, Olivia Kyriacou, Janet Treasure

**Affiliations:** 1 Department of Psychological Medicine, Section of Eating Disorders, King's College London Institute of Psychiatry, London, UK; 2 Department of Psychology, Institute of Psychiatry, King's College London, UK

## Abstract

**Background:**

Families of people with eating disorders are often caught up in rule bound eating and safety behaviours that characterise the illness. The main aim of this study was to develop a valid and specific scale to measure family accommodation in the context of having a relative with an eating disorder.

**Methods:**

A new scale, the Accommodation and Enabling Scale for Eating Disorders (AESED), was jointly generated by professionals and expert carers through qualitative analysis. In the first stage, this instrument was given to 201 family members of relatives diagnosed with an eating disorder, with additional self-report measures including the Experience of Caregiving Inventory (ECI), the Hospital Anxiety and Depression Scale (HADS) and the Family Questionnaire (FQ). In the second stage, the sensitivity of the AESED to change was tested in a pre-and-post design study with a new sample of 116 caregivers, using a DVDs-distance skills training for caregivers.

**Results:**

A 33 item instrument was derived consisting of five factors: Avoidance and Modifying Routine, Reassurance Seeking, Meal Ritual, Control of Family and Turning a Blind Eye, which together explained 60.1% of the variance. This scale had good psychometric properties in terms of Cronbach's alpha which ranged from 0.77 to 0.92. Regarding the convergent validity, most of the AESED subscales was moderately supported by correlations with anxiety (HADS; r = 0.24 to 0.48) and depression levels (HADS; r = 0.17 to 0.47), negative caregiving (ECI; r = 0.18 to 0.45), and expressed emotion levels (FQ; r = 0.17 to 0.51). Pre-post intervention assessments showed that the overall AESED scale (*d *= 0.38) and the avoidance and modifying routine (*d *= 0.52), meal ritual (*d *= 0.27) and control of the family (*d *= 0.49) subscales were sensitive to change.

**Conclusion:**

Internal consistency was good and initial validity of the scale was adequate, it was able to discriminate differences between clinical variables, however, further work is needed to confirm the factor structure and validity of the AESED. Nevertheless, this scale may be of value in exploring and helping to improve carers' coping strategies and in examining the effectiveness of family based interventions.

## Background

Eating disorders (ED) have a considerable impact on affected families' lives. Clinical evidence suggests that family members of relatives with an ED suffer significant emotional strain and these families are often characterised by fraught and distressing patterns of interpersonal interaction [1-5]. Interpersonal issues form one of the core perpetuating processes in Schmidt and Treasure's model of maintenance of anorexia nervosa [6]. Caregivers of ED patients have reported poor quality of life and their role is associated with high subjective burden of care, anxiety, depression, loss of behavioral or emotional control, and low psychological well-being [7-10].

It has been suggested that families may accommodate patients' symptoms in attempts to alleviate family conflict and stress [5,11,12]. Accommodation of symptoms by families may negatively impact on sufferer outcomes. It is thought that by tolerating or allowing symptomatic behaviours to continue they may gradually become reinforced or even endorsed within the family context as caregivers become increasingly entrapped within the rule bound eating, weight and shape control behaviours that characterise the illness. Many families accommodate to these symptoms by trying to avoid feelings of helplessness and anger in disputes during mealtimes or may offer reassurance to the patient, thus organising family life around the illness. Assuming this accommodating role, caregivers display a range of emotional responses from guilt and self-blame, to anger and disgust, as well as high levels of anxiety and frustration for accepting these problematic behaviours and their impact on family functioning [13]. Hence, high levels of expressed emotion (EE) such as critical comments directed towards their sufferer, intensifies conflicts within family and this has been shown to have a negative influence on treatment outcome [14]. In contrast, there appears to be an association between greater familial distance in terms of living arrangements and carer well-being [15].

Dysfunctional family functioning is associated with a greater level of eating pathology (comorbidity, long-term duration of the illness, long-term dependency) as well as with more distressing caregiving experience [1,16]. Additionally, people with ED characteristically display obsessive compulsive traits such as rigidity, ritualism, perfectionism, and meticulousness in their behaviours and thoughts [17]. Family members report giving into the ED relatives' rules associated with eating, such as accepting the use of kitchen scales for weighing out portions, cutting solid food into minuscule pieces and allocating the patient's daily ration in small containers. In summary, families can be drawn into organising their life around eating disorder behaviours and accommodate to or enable some of the core symptoms [18]. However the assessment of this aspect of family functioning has not been thoroughly examined in relatives with eating disorders and a validated instrument has yet to be developed.

Families caring for an individual with an obsesive-compulsive disorder (OCD) are frequently characterized by compulsive behaviors, obsessive thinking and perfectionism. These families present similarities with eating disorder caregivers in how they respond to their relative's illness, as well as how they also accommodate symptoms in attempts to alleviate family strain, stress or distress [19,20]. Hence, an OCD spectrum may provide a good conceptual model due to its suggested overlap with eating disorders. Likewise, the concept of accommodation has already been developed and assessed in OCD caregivers, which is a useful starting point for a study of this nature. Calvocoressi and colleagues [21] have developed an instrument to formally measure this accommodation. The Family Accommodation Questionnaire (FAS) assesses the extent to which relatives of patients with an OCD engage in different types of accommodating behaviours, namely reassuring patients about their obsessions, refraining from saying things that might trigger symptoms, facilitating patient avoidance, participating in the patient's rituals, assisting patients with simple tasks or decisions, modifying work, family or social routines because of the patients' needs, or tolerating aberrant behaviours at home. Thus, it appears that the level of family accommodation is closely related to impaired parental functioning [7,22,23]. Moreover it appears that the level of family accommodation likewise plays an important role in the patient's response to their treatment [24-26]. As we believe that there are apparently considerable similarities between the behaviours and thoughts of carers of relatives with an OCD, as captured by the FAS, and the behaviours and thoughts of carers of relatives with an ED, we were interested in building on and adapting the concepts assessed for people with an OCD to the families of people with eating disorders.

Skills training programmes specifically designed to improve coping skills for families have been associated with a reduction of levels of caregiver burden and psychological distress [10,27,28]. These family-based interventions appear to be effective for ED caregivers and it is therefore important to have appropriate instruments capable of assessing further caregiving aspects such as carers' level of accomodation to ED behaviours. Furthermore, we believe that this instrument will allow us to examine possible changes in caregivers' ability to cope with stressful situations and gauge possible improvements in their caregiving roles.

The purpose of the present study was to undertake four aims: (1) to develop and validate a new scale, designed to measure accommodating and enabling behaviour by carers of relatives with an eating disorder; (2) to examine whether these behaviours are related to high depression and/or anxiety levels, to high expressed emotion and to negative caregiving aspects in the primary caregiver; (3) to examine whether the degree of family involvement in the patients' rituals is related to clinical or psychosocial features such as type of diagnosis, co-morbidity, the amount of weekly face-to-face contact between patient and carer, and any past ED history of the carer; and (4) to examine whether the instrument is sensitive to change.

## Method

### The development of the Measure

We used some of the general items from the *Family Accommodation Questionnaire *(FAS;[21]) in conjunction with specific eating disorder items that we have developed to capture all of the family accommodating behaviours. Permission to use or modify statements to ensure their relevance to eating disorders symptoms was given by the first author, Dr. Lisa Calvocoressi.

The FAS measures how relatives of patients with OCD engage in 12 types of accommodating behaviours and it is administered as an interview by a clinician or trained interviewer. These include the nine accommodating behaviours assessed by the original questionnaire (ie. reassure patients regarding the unfounded nature of their obsessions) and three new items related to the rituals (FAS-IR; Calvocoressi et al., 1999). Each item is scored on a scale ranging from 0 (i.e., None/Not at all) to 4 (i.e., Everyday/Extreme). Total FAS scores range from 0 to 48 and are obtained by adding the item scores. The scale has good internal consistency (alpha = 0.76) for the first 9 original items; [21]. Cronbach's alpha coefficient for the total 12 items was 0.82 [19].

The items for the Accommodation and Enabling Scale for Eating Disorders were generated by a panel of clinicians and researchers using transcripts of recordings from a series of pilot carers' workshops and earlier quantitative and qualitative work with families [2,4,11,15,29]. The panel was made up of two psychiatrists (one of the authors of this paper, JT) currently working at the South London and Maudsley Hospital (SLaM) as well as three PhD level research psychologists in Psychology (two of whom are also co-authors of this paper, AR and OK). All of the panel members were working at the Eating Disorder Unit (EDU) at the time of the study. The items were reviewed by two "expert carers" (two mothers of daughters with eating disorders) associated with our Unit. These two mothers have run carer support groups in the London region and have also collaborated closely with the EDU in previous research. Following several in-depth discussions by the panel, a total of 41 statements were established based on criteria of clarity, relevance and significance for field-testing using a 5-point Likert scale (where 0 = never, 1 = rarely, 2 = sometimes, 3 = often, 4 = nearly always). Item 28 was modified into a Visual Analogue Scale (VAS) from 0 to 10, after feedback from the two expert carers who found it difficult to give a reliable answer to this item: "In general, to what extent would you say that the relative with an eating disorders controls family life and activities?" Items referred to observations and experiences of the past month and are shown in Additional File 1.

Specifically, we developed 35 new items through our panel of clinicians and we used 6 items from the FAS-IR (AESED: items 29, 30, 31, 33, 34 and 35 (see Additional File 1)). Thus, a total of 41 items were tested.

### Subjects and procedure

Recruitment and assessments were completed in two stages: in the first stage, and over a period of two years. Carers were given information regarding the assessment instruments and were asked about their interest in possible family interventions at the Unit (n = 201). In the second stage and over a period of 18 months, a new set of participants were recruited from an ongoing study to participate in a novel DVD skills-based distance intervention programme (n = 116).

The participants in the first stage had been recruited to take part in a parallel study and were therefore excluded from the second stage. Caregivers for both stages were recruited from our research website , from B-eat (*B-eat *is a national charity based in the UK providing support and help for people with eating disorders and their families) and from carer support groups in the UK. B-eat includes carers of people who are in treatment and people who are not in treatment. Several specific groups of caregivers were included: 1) caregivers who had prior experience of a family intervention and who had expressed the need for more help, 2) caregivers of people admitted to the inpatient unit with no previous experience of carer work, 3) caregivers of patients currently in the outpatient eating disorder and 4) caregivers of people either not in current treatment or in services elsewhere (recruited from the Institute of Psychiatry website and *B-eat*). To be eligible for the study, the caregiver had to be either living with, or directly involved in the care of someone with an eating disorder. The exclusion criterion regarding incomplete questionnaires was set at three or more incomplete items (n = 8). Only primary caregivers in this first stage completed a pack of self-report questionnaires (HADS, ECI, FQ and 41-original AESED). Following these guidelines, questionnaires from 193 caregivers were included in the exploratory factor analysis, reliability and convergent analysis. Ethical committee approval was granted for the study (Ref. No. 238/04).

These samples for both stages were self-selected and we are unable to accurately comment on the number of caregivers who chose not to access information about the study. Also, we cannot provide reasons as to why, in either stage, caregivers chose not to participate in the intervention.

In the second stage, the participants were part of an ongoing study to assess the DVD programme. The participants from the DVDs skills-based distance intervention study completed a pack of self-report questionnaires (HADS, ECI, FQ described below) and the final version of the AESED once a written informed consent was obtained. Ethical committee approval was extended for the next stage of the study by the Institute of Psychiatry (Ref. No. 238/04). The post-intervention assessment was collected after the intervention (9 weeks later). The time interval between the first and second administration was the same across participants. The aim of the DVD-based training was to equip caregivers with the skills and knowledge needed to support and encourage those suffering from an eating disorder and to help them to break free from the traps that prevent recovery. The content of the intervention has been previously described elsewhere [10,20,30]. A comprehensive manual [30] accompanied the DVDs. The results from a previous pilot study suggested that caregivers expressed high levels of satisfaction with most aspects of the training [31]. A total of 116 caregivers agreed to take part in this study until January 2008. The recruitment stage finished in May 2009. The secondary caregivers were excluded from statistical analysis due to the problem of lack of independence, and then 106 caregivers were included for pre-post intervention analysis.

The questionnaires mentioned in the first stage were also used as validity measures for the AESED as they assess general aspects of caregiving and psychological morbidity. The validation analyses described below were conducted using the final version of AESED.

### Measures

The *Hospital Anxiety and Depression Scale HADS *[32,33] is a 14-item instrument designed to detect the presence and severity of anxiety and depression. The scoring for both subscales ranges from 0-21. The subscales have shown high internal consistency (0.80 to 0.93 for anxiety and .81 to .90 for depression). A score of 11 or higher for each subscale is indicative of the corresponding mood disorder.

The *Experience of Caregiving Inventory ECI *[34] is a measure of stress, appraisal, and coping in carers of an individual with a severe mental illness. The ECI is a 66 item self-report questionnaire (using a Likert scale method scored from 0 to 4). A total of eight subscales (Difficult Behaviours, Negative Symptoms, Stigma, Problems with Services, Effects on Family, Need to Backup, Dependency and Loss) measure negative aspects of caregiving and have reliability ranging between 0.74 and 0.91. A higher score indicates more negative appraisals (ECI-negative; ranges from 0 to 208). There are also two positive scales, Positive Personal Experiences and Good Relationship with the Patient, that measure positive aspects of caregiving. Higher scores indicate positive appraisals (ECI-positive; range from 0 to 56). However, for the validation purpose of this study only the overall score on the ECI-negative dimension was used in order to isolate specific negative aspects of caregiving in eating disorders.

The *Family Questionnaire FQ *[35] consists of 20 items measuring expressed emotion (EE), (10 for criticism (CC) and 10 for emotional over-involvement (EOI). The scoring ranges from 1 as "never/rarely" to 4 as "very often" and a higher total score indicates higher expressed emotion. The FQ has good internal consistency (ranging from 0.78 to 0.80 for emotional over-involvement (EOI) and from 0.91 to 0.92 for criticism (CC). The original authors provide a cut-off point of 23 for CC as an indication of high criticism, and 27 for high EOI.

### Statistical analysis

In the first stage, the following analyses were conducted to test the psychometric properties of the Accommodation and Enabling Scale (n = 201).

#### Principal Component Analysis

An exploratory factor analysis (PCA) was performed using the principle component extraction method with Varimax rotation using SPSS.13. An Eigenvalue >1, was used to retain possible factors/subscales. Only items that loaded at 0.40 or higher on the factor matrix were selected. The Kaiser-Meyer-Olkin (KMO) measure of sampling adequacy and Barlett's Test of Sphericity are reported for assessing the factor structure of the data. A screeplot and Monte Carlo technique after PCA for Parallel Analysis were then conducted to determine the number of factors to retain.

#### Reliability

The internal consistency of the subscales was assessed by measuring Cronbach's alpha for the total scale and item-scale. Item-total subscale correlations were calculated for ED caregivers.

#### Convergent and discriminant validity

Convergent and discriminant validity was established by correlating the Accommodation and Enabling Scale for Eating Disorders (AESED) with the 5 factors scores and ECI-negative dimension, HADS-depression and HADS-anxiety, FQ-CC and FQ-EOI and with demographic and clinical variables. Spearman correlation was used as four subscales of the AESED were skewed (non-normal distribution) and Mann-Whitney U tests were used due to categorical variables. Distributions of the items and subscales scores are reported in terms of range, means, and standard deviation. Categorical variables were: type of diagnosis (anorexia/bulimia nervosa), comorbidity with drug/alcohol abuse, self-harm or gambling (yes/no) and caregivers with their own lifetime history of eating problems (current obesity or anorexia nervosa or bulimia nervosa not diagnosed when younger) (yes/no). The average number of contact hours was converted to a binary measure (less than 21 h/w or more than 21 h/w).

In the second stage, the following analyses were conducted to test the sensitivity of the Accommodation and Enabling Scale (n = 116).

#### Responsiveness

Wilcoxon's signed-ranks test for paired samples and chi-square test were used to assess change following the caregiver intervention. Effect sizes were calculated using Cohen's *d *to indicate the magnitude of pre and post differences. The guidelines for interpreting this value (*d*) are: < 0.4 = small effect, > = 0.4 = moderate effect, > = 0.75 = large effect [36].

## Results

### Demographic data

For the first stage, of the 201 primary caregivers that requested information and completed the battery of questionnaires, only 193 returned fully completed questionnaires (see Table 1). One hundred and sixty-seven caregivers (85.5%) were females. The average age of the caregivers was 49.6 years (SD = 8.4). Forty-two (25.3%) of the carers were educated up to secondary level and 121 (62.7%) were educated to higher education; the remaining thirty gave no information on their education (12%). One hundred and seventy-six carers (91.0%) were parents. One hundred and fifty carers (78.0%) were currently living with the patient. The patients were 98% females with a mean age of 21.3 years (6.8). Clinical symptoms reported by the carer were as follows: 90% (n = 147) restricted food intake, 49.4% (n = 80) exercised excessively, 34.2% (n = 55) vomited, 26% (n = 42) binged and 10.5% (n = 17) stole food/money for binges. Eighty-seven (45%) carers reported comorbid impulsive behaviours (alcohol abuse, illegal substances, self-harm or gambling) in patients.

**Table 1:** Demographic details of carers and patients from the two studies

	**1^st ^stage. Carers for Assessment**			**2^nd ^stage. Carers Intervention**		
	
	**N**	**%**	**Mean(SD)**	**N**	**%**	**Mean(SD)**
*Carers*	193			116		

Age	-	-	49.6 (8.4)			50.1(8.3)
Sex						
Male	26	14.5		30	25.8	
Female	167	85.5		86	74.2	
Marital status						
Married/living together	142	69		95	81.9	
Single/Divorced/separated	51	31		21	18.1	
Highest education level						
School/Secondary Level	42	25.3		38	32.8	
Degree/Diploma level	121	62.7		78	67.2	
Employment status						
Full/Part time	115	60.3		85	73.3	
Not employed	78	39.7		31	26.7	
Relationship with sufferer						
Parents	166	91		107	92.2	
Husband/Partner	10	5.2		7	6	
Sibling/Friend	7	3.8		2	1.8	
Living with patient						
Yes	150	78		87	75	
Amount of contact with patient						
< 21 hours/w						
> 21 hours/w	77	39.4		52	44.8	
	116	60.6		64	55.2	

Had had previous eating not diagnosed						
	47	24%		39	33.6	

*Patient*	193			106		
Age	-	-	21.3 (6.8)			21.6 (7.2)
Sex						
Male	4	7		3	2.8	
Female	190	98		103	97.2	
Diagnosis (carers' report)						
Anorexia	136	70.5		88	83	
Bulimia	47	24.5		7	6.6	
Unclear Diagnosis	10	5		11	10.4	

In the second stage, a total of 194 caregivers from an ongoing study requested information of which 116 caregivers (60%) agreed to participate in the intervention and were given the final version of the AESED (scale of 33 items). The data is shown in Table 1. Ten participants who were secondary caregivers for the same relative were not included in the analysis, due to the problem of lack of independence of observations and because the emphasis was on primary caregivers and therefore more involved carers. Overall, from 106 primary caregivers who participated in the intervention a total of 89 completed the pre and post intervention assessments (84% response rate). Seventeen carers did not complete the post-intervention assessment. We were not able to compile information as to why they did not complete this assessment.

### Factor analysis of the Accommodation and Enabling Scale (AESED)

The first exploratory factor analysis was performed on the 41 items from the original instrument. A ten-factor structure was derived, explaining 70% of the total variance. Following a preliminary appraisal of the results, a principal components analysis was conducted excluding items with a factor loading lower than 0.4 on any individual factor and those items that loaded equally on more than one factor. This revealed a five-factor solution with 33 items accounting for 60.1% of the variance. The KMO measure of sampling adequacy was 0.82, exceeding the recommended 0.6 and Barlett's Test reached statistical significance (p < 0.01). An inspection of the screeplot revealed a clear break after the fifth component. This was further supported by the results of Monte Carlo PCA for Parallel Analysis, which showed only five components with eigenvalues exceeding the corresponding criterion values. These 5 factors were interpreted as themes of avoidance and modifying family routine, reassurance seeking, meal ritual, control of family and turning a blind eye. Table 2 shows the item loadings, variance explained, item-total correlations by subscales, communality values and reliabilities for these five subscales. Communality values that demonstrated how well items' variance was explained by the five-factor solution ranged from 0.35 to 0.80. These values were above 0.30 and indicated that the variance of each item was adequately explained by the five-factor solution [37].

**Table 2:** Principal Component Factor Analysis with Varimax rotation for a 5-factor solution of AESED for the carers of relatives with an eating disorder (1^st ^stage; N = 193)

	**Factor Loadings**					**Item-scale correlation**	**Communality**
**Items**	**1**	**2**	**3**	**4**	**5**		

*Factor 1:Avoidance &Modifying Routine* *(AMR) (Cronbach alpha 0.90)*							

38. Has helping your relative in the previously mentioned ways caused you distress?	**0.79**	0.12	0.10	-	-	0.56	0.65
34. Have you modified your family routine because of your relative's symptoms?	**0.78**	-	0.17	0.15	-	0.60	0.67
37. Have you modified your leisure activities because of your relative's needs?	**0.77**	0.16	-	0.15	0.11	0.70	0.65
33. Have you avoided doing things, going places or being with people because of your relative's disorder?	**0.76**	0.16	-	0.26	-	0.61	0.68
39. Has your relative become distressed when you have not provided assistance?	**0.75**	0.23	0.10	-	0.15	0.64	0.66
36. Have you modified your work schedule because of your relative's needs?	**0.69**	0.11	-	-	-	0.45	0.50
40. Has your relative become angry/abusive when you have not provided assistance?	**0.68**	0.27	0.11	-	0.18	0.62	0.59
32. How often did you assist your relative in avoiding things that might make him/her anxious?	**0.65**	-	0.18	-	0.11	0.49	0.47
28. To what extent would you say that the relative with an eating disorder controls family life and activities?	**0.64**	0.23	0.13	0.25	-	0.64	0.55
31. How often did you participate in behaviours related to your relative's compulsions? Over the past week?	**0.45**	-	0.26	0.11	0.31	0.48	0.39

*Factor 2: Reassurance Seeking(RS)* *(Cronbach alpha 0.86)*							

9. Repeated seeking of reassurance about whether she looks fat in certain clothes?	-	**0.84**	-	-	0.11	0.43	0.73
7. Repeated questioning about whether she will get fat?	0.13	**0.83**	-	0.13	-	0.45	0.72
8. Repeated questioning whether it is safe or acceptable to eat certain foods?	0.17	**0.75**	0.13	0.16	-0.15	0.51	0.66
22. Accommodation of routines of checking their body shape or weight?	-	**0.67**	0.20	-	0.10	0.45	0.52
13. Repeated conversations about negative thoughts and feelings?	0.17	**0.67**	-	0.14	-	0.48	0.50
14. Repeated conversations about self-harm?	0.27	**0.63**	-	-		0.43	0.40
12. Repeated conversations about ingredients and amounts in food preparation	0.25	**0.54**	0.28	0.19	-0.26	0.52	0.54
21. Accommodation of the exercise routine of the relative with an ED?	0.11	**0.48**	0.33	-	-	0.40	0.35

*Factor 3: Meal Ritual(MR)* *(Cronbach alpha 0.86)*							

19. Accommodating to how the kitchen is cleaned?	-	-	**0.89**	-	-	0.38	0.80
16. Accommodating to how crockery is cleaned?	-	-	**0.84**	-	-	0.43	0.72
15. Accommodating to what crockery is used?	0.18	0.13	**0.70**	0.21	-	0.53	0.58
20. Accommodating to how food is stored?	0.13	-	**0.68**	0.14	-	0.43	0.50
18. Accommodating to what place food is eaten in?	0.32	-	**0.65**	0.31	-	0.59	0.64
23. Accommodating to how the house is cleaned and tidied?	-	0.20	**0.62**	-	0.28	0.40	0.50
17. Accommodating to what time food is eaten?	0.24	0.14	**0.53**	0.32	-0.24	0.48	0.52

*Factor 4: Control of Family(CF)* *(Cronbach alpha 0.85)*							

3. Control cooking practice and ingredients used	0.28	0.18	0.14	**0.78**	-	0.55	0.75
4. Control what other family members eat	-	0.22	0.23	**0.78**	-	0.51	0.72
2. Control what family members do and for how long in the kitchen	0.18	-	0.38	**0.72**	-	0.53	0.72
1. Control choice of food that you buy	0.35	0.14	-	**0.71**	-	0.55	0.65

*Factor 5: 'Turning a Blind Eye' (TBE)* *(Cronbach alpha 0.77)*							

27. Ignore bathroom left in a mess	0.14	-	-	-0.13	**0.76**	0.14	0.62
24. Ignore food disappearing	0.13	-	-	0.25	**0.76**	0.32	0.66
26. Ignore kitchen left in a mess	0.18	-	-	0.25	**0.72**	0.29	0.62
25. Ignore if money is taken	0.13	-	-	-0.13	**0.65**	0.19	0.47

**Eigenvalue**	9.5	3.4	3.0	2.2	1.7		
**Percentage Variance explained**	28.7	10.3	9.1	6.6	5.3		
Cumulative percentage variance explained	17.3	30.3	43.1	52.2	**60.1**		

*Note. Bold values showing the five factor loadings

*Note. Loadings below 0.10 are not presented.

Table 2 shows the 33 statements chosen for the AESED. Eight items, 5, 6, 10, 11, 29, 30, 35 and 41 were deleted after the principal components analysis.

### Scoring the AESED

A total rating for each factor was computed for the main analysis, by adding the scores of the items belonging to a specific domain (avoidance and modifying routine, reassurance seeking, meal ritual, control of family and turning a blind eye). The score range for each item was from 0 to 4: scale score ranges for subscale dimensions varied according to the number of items of the subscale. Scores were computed as a 5-point Likert scale in which 0 and 1 was 0; 2 and 3 was 1; 4, 5 and 6 was 2; 7 and 8 was 3; and 9 and 10 was 4 for item 28, consisting of a Visual Analogue Scale (VAS). This scoring was done to allow for greater discrimination of caregivers' answers. The inclusion of the item 28, improved item homogeneity and overall scale and subscale reliability. Additionally, a total score was calculated in two ways a) by summing up the scores of all the items in order to obtain an overall score of family accommodation to eating disorder symptoms and b) by computing a total score by adding the mean domain scores of each subscale. As the two methods gave scores which correlated >0.9, we concluded that the overall AESED score would be obtained by summing up the unweighted scores of all the items: the total scale is therefore scored from 0 to 132. A higher score is associated with higher family accommodation to eating disorder symptoms.

### Reliability

Cronbach's alpha values for each of the subscales of the Accommodation and Enabling Scale were: 0.90 for the Avoidance & Modifying Routine subscale, 0.86 for Reassurance Seeking subscale, 0.86 for the Meal Ritual subscale, 0.85 for Control of Family subscale and 0.77 for Turning a Blind Eye. The value for the total instrument was 0.92 (see Table 2).

### Item-total subscale correlations and intercorrelation

Correlational analyses between items and total subscales were conducted to measure the degree with which the items for each subscale capture specific characteristics or homogeneity as shown in the Table 2. The overall Accommodation and Enabling Scale score item-scale correlations ranged from 0.14-0.70. Item-scale correlation ranged from 0.48 to 0.70 in the Avoidance & Modifying Routine subscale, 0.40 to 0.52 in the Reassurance Seeking subscale, 0.38 to 0.59 in the Meal Ritual subscale, 0.51 to 0.55 in the Control of Family subscale, and finally, 0.14 to 0.32 in the Turning a Blind Eye subscale.

There were strong and significant correlations between the subscales scores and the total score of AESED (except for the Turning a Blind Eye subscale) with correlations ranging between 0.68 and 0.85 (Table 3). However, moderate and significant correlations were found between subscales scores ranging from 0.30 to 0.51 (p < 0.01), except Turning a Blind Eye subscale scores which showed low associations with most of the subscale scores.

**Table 3:** Correlations (Spearman) between the Accommodation and Enabling Scale (AESED) subscales scores and ECI-negative, HADS, FQ (1^st ^stage; N = 193)

**ED subscales**	**Reassu**. **Seeking** **(RS)**	**Meal Ritual** **(MR)**	**Control Family** **(CF)**	**T. Blind Eye** **(TBE)**	**Overall** **AESED**	**ECI-negative**	**HADS-Depress**.	**HADS-** **Anxiety**	**FQ-CC** **FQ-EOI**
*Avoidance & Modifying R.*	0.43**	0.44**	0.46**	0.30**	0.85**	0.45**	0.47**	0.48**	0.51** 0.44**
*Reassure Seeking*	-	0.33**	0.31**	0.11	0.69**	0.33**	0.17*	0.30**	0.17* 0.15
*Meal Ritual*	-	-	0.51**	0.09	0.68**	0.18*	0.31**	0.16	0.18* 0.17*
*Control of Family*	-	-	-	0.17*	0.69**	0.24**	0.34**	0.26**	0.26** 0.18*
*'Turning a Blind Eye'*	-	-	-	-	0.33**	0.36**	0.11	0.24**	0.31** 0.21*

***Overall AESED score (33 items)***	-	-	-	-	-	0.43**	0.45**	0.48**	0.49** 0.40**

Note. ** Correlation is significant at the 0.01 level (2-tailed).

Note.* Correlation is significant at the 0.05 level (2-tailed).

Note. Correlations with AESED scale are presented in bold style.

### Convergent and Discriminant Validity of the Accommodation and Enabling Scale for Eating Disorders (AESED)

The convergent and discriminant validity was studied by correlating the four subscales scores and the total score of the Accommodation and Enabling Scale with the level of negative appraisals measured by the ECI, the level of depression and anxiety measured by the HADS and with the level of expressed emotion measured by the FQ. The results are illustrated in Table 3. There were positive associations between negative appraisals of caregiving (ECI), AESED total score and subscale scores, HADS-depression and HADS-anxiety. All dimensions of the Accommodation and Enabling Scale (AESED) were significantly related to EE (CC and EOI) measured by the Family Questionnaire (Spearman's rho = 0.49 for EE-CC and Spearman's rho = 0.40 for EE-EOI, n = 130, p < 0.01). The subscale of AESED that had the strongest correlation with the different instruments was the Avoidance and Modifying Routine (Spearman's rho > 0.45, p = 0.01).

The Accommodation and Enabling Scale (AESED) scores by clinical and psychosocial features are shown in Table 4. The Control of Family subscale was particularly high in carers with a relative with anorexia nervosa whereas the Turning a Blind Eye subscale was high in carers with a relative with bulimia nervosa. Impulsive behaviours (alcohol abuse, illegal substances, self-harm or gambling) in the ill relative were associated with high scores on the Reassurance Seeking and Turning a Blind Eye subscales. These behaviours were associated with caregiving burden and criticism.

**Table 4:** Carers' AESED scores by eating disorder diagnosis, co-morbidity, average number of contact hours a week and carers own eating problems (1^st ^stage; n = 193)

**Variables**	**Avoidance & M**.	**Reassuranc S**.	**Meal Ritual**	**Control Family**	**T. Blind Eye**	**Overall AESED**	**ECI-Negative**	**FQ-CC**	**FQ-EOI**
**Total sample**	19.7 (9.8)	11.2 (7.8)	7.7 (7.1)	8.5 (4.5)	2.3 (3.5)	49.4 (23.3)	103.9 (36.6)	23.4 (5.5)	28.2 (4.4)

**AN carers (N = 136)**	19.9 (10)	11.3 (8.0)	8.1 (7.4)	8.9 (4.3)	1.7 (3.2)	49.8 (23.2)	103.1 (35.2)	23.4 (5.6)	28.5 (4.2)
**BN carers** **(N = 47)**	19.0 (9.8)	10.9 (7.6)	6.1 (6.2)	7.3 (5.1)	4.2 (3.8)	47.2 (23.3)	110.3 (37.4)	23.3 (5.5)	27.4 (4.7)

**p-value** **(2-tailed)**	**n.s**.	**n.s**.	**n.s**.	**0.046**	**0.001**	**n.s**.	*n.s.	*n.s.	*n.s.

**Co-** **Morbidity+ (N = 87)**	21.1 (9.6)	12.3 (7.8)	7.4 (7.2)	8.1 (4.7)	2.8 (3.6)	51.6 (22.6)	112.5 (34.6)	24.6 (5.6)	28.3 (4.6)
**Non Co-** **morbidity** **(N = 62)**	19.2 (9.1)	9.1 (6.7)	6.8 (6.3)	8.6 (4)	1.7 (3.1)	45.2 (20)	91.2 (36.9)	22.1 (5.5)	27.7 (4.2)

**p-value** **(2-tailed)**	**n.s**.	**0.018**	**n.s**.	**n.s**.	**0.035**	**n.s**.	*0.001	*0.01	*n.s.

**less 21 hours/w** **(N = 70)**	16.9 (9.1)	9.3 (7.9)	5.7 (6.2)	6.8 (4.8)	1.9 (3)	41.2 (21.2)	100.8 (33.2)	22.8 (5.6)	26.7 (4.3)
**more 21 hours/w** **(N = 106)**	21.0 (10.3)	12.2 (7.6)	8.4 (7.2)	9.6 (4)	2.4 (3.5)	53.0 (23.6)	102.6 (38.4)	23.3 (5.3)	28.6 (4.2)

**p-value** **(2-tailed)**	**0.012**	**0.009**	**0.010**	**0.001**	**n.s**.	**0.005**	*n.s.	*n.s.	*0.01

**Mother** **previous eating problem** **++ (N = 47)**	21.6 (9.9)	12.0 (7.8)	9.0 (8.4)	9.1 (4.4)	3.6 (3.7)	55.9 (22.9)	114.9 (31)	25 (5.5)	29.9 (3.9)
**Mother without EP(N = 94)**	19.5 (9.6)	10.2 (7.7)	5.8 (5.9)	8.0 (4.6)	1.6 (3)	44.6 (21.8)	100.5 (38)	22.6 (5.4)	27.5 (4.5)

**p-value** **(2-tailed)**	**n.s**.	**n.s**.	**0.052**	**n.s**.	**0.002**	**0.032**	*0.04	*0.02	*0.01

Note. Data are shown as means (SD = standard deviation).

Statistical comparison is shown between scores groups using the Mann-Whitney test.

* Statistical comparison is shown between scores groups using the t-test.

+ Comorbidity-impulsive behaviours regard to alcohol abuse, illegal substances, self-harm or gambling

++Eating problem-their own lifetime history of eating problem (past/current obesity or anorexia nervosa or bulimia nervosa not diagnosed/diagnosed)

Caregivers who had more than 21 hours a week contact with the patient presented higher scores in the overall AESED, as well as on individual subscales of Avoidance and Modifying routine, Reassurance Seeking, Meal Ritual and Control of the Family. These family members also presented higher levels of expressed emotion at home, specifically in terms of emotional over-involvement.

Caregivers with their own eating problems had a higher overall accommodation (AESED) index, reporting more difficulties with meal rituals and a higher tendency to ignore the negative consequences of the patient's symptoms (turning a blind eye subscale). These carers also experienced high caregiving burden with more criticism and over-involvement (Table 4).

### Responsiveness to change

Following the caregivers' intervention the overall AESED index score was reduced (small effect size) and a moderate sized improvement was found on the avoidance and modifying routine (AMR) and the control of the family (CF) subscales (Z = -4.3 and Z = -3.8 p < 0.01, Wilcoxon's signed-ranks test for paired samples) (see Table 5). This improvement paralleled the reduction in anxiety and depression levels (HADS), caregiving burden (ECI) and expressed emotion (over-involvement), (FQ) following the intervention.

**Table 5:** DVDs-skills-based intervention effect from means and standard deviations pre- to post-intervention from HADS, ECI, FQ and AESED scores.

**Variables**	**N**	***Baseline*(*T*_1_)** ***Means (SD)***	***Post-interv*(*T*_2_)** ***Means (SD)***	***Z***	***p-value*** ***(2-tailed)***	***d***
*Primary Outcome*	89					

**Hospital Anxiety and Depression Scale (HADS)**						
***Depression scale(0-21)***	82	7.5 (4.4)	6.0 (4.6)	-3.8	0.01	***0.34***
***Anxiety scale (0-21)***	80	12.0 (4)	10.1 (4.5)	3.6	0.01	***0.47***

*Secondary Outcome*						

**Experience of Caregiving Inventory (ECI)**						
***ECI-Negative(0-208)***	74	102 (37.7)	83 (37.5)	-5.3	0.01	***0.51***

**Family Questionnaire(FQ)**						
***Criticism (10-40)***	85	23.3 (5.5)	22.3 (5.8)	-1.0	0.30	***0.20***
***Emotional Over-involvement (10-40) ***	67	28.4 (4.4)	26 (4.4)	-5.0	0.01	***0.55***

**Accommodation and Enabling Scale (AESED)**						

***Avoidance & Modifying R.*** ***(0-40)***	76	20.6 (9.7)	15.6 (9.7)	-4.3	0.01	***0.52***

***Reassure Seeking (0-32)***	75	9.4 (7.4)	8.8 (6.9)	-0.9	0.35	***0.08***

***Meal Ritual (0-28)***	82	7.7 (7.6)	5.8 (6.4)	-2.4	0.01	***0.27***

***Control of Family (0-16)***	79	8.0 (4.8)	5.7 (4.6)	-3.8	0.01	***0.49***

***'Turning a Blind Eye' (0-16)***	71	2.6 (3.4)	2.3 (3.6)	-0.9	0.34	***0.10***

***OVERALL score(0-132)***	70	48.3 (24)	39 (25)	-3.8	0.01	***0.38***

*d = Effect size was calculated on based of subscales' means and standard deviations

Wilcoxon's signed-ranks test for paired samples was used for non-parametric distribution (2^nd ^stage; n = 106)

Over 61.3% of caregivers (n = 87) scored at or above the clinical threshold (score> = 11) for HADS-anxiety at baseline compared with 48.3% (n = 43) after the intervention (*X*^2 ^= 6.4, df = 1, p = 0.011), and 20.1% (n = 29) scored at or above the clinical threshold for HADS-depression compared with 17% (n = 15) following the training (*X*^2 ^= 13.3, df = 1, p = 0.001). Regarding cut-off point for high EE, 56% of carers (n = 87) scored at or above 23 for high-CC at baseline compared with 50% (n = 45) after the intervention (*X*^2 ^= 31.4, df = 1, p = 0.001), and 65.5% (n = 100) scored at or above 27 for high-EOI at baseline compared with 49.3% (n = 36) following the training (*X*^2 ^= 17.9, df = 1, p = 0.001).

## Discussion

The primary aim of this study was to develop and validate a new measure, the Accommodation and Enabling Scale for Eating Disorders (AESED), designed to measure accommodating and enabling behaviours by families/caregivers of relatives with eating disorders. We found that an instrument with 33 items and five factors, Avoidance and Modifying Routine, Reassurance Seeking, Meal Ritual, Control of Family and Turning a Blind Eye, encapsulated the accommodating behaviours and thoughts that were expressed by caregivers.

The conceptualisation of the instrument was inspired by the seminal work in the families of people with OCD by Calvoressi and colleagues [19,21]. The specification to eating disorders was derived from qualitative and quantitative work in our department [2,4,11,15,29]. Although the FAS has demonstrated a good internal consistency (α = 0.82), our scale obtained higher alpha values for the overall scale (α = 0.92).

We found moderate correlations between scores on the AESED scale and caregiver depression and/or anxiety levels, caregiving burden and expressed emotion measures. In contrast to caregivers of people with OCD the FAS scores were not associated with expressed emotion as measured by the FMSS (Five Minute Speech Sample [38]) [19]. This difference may suggest that EE is a more relevant construct in ED and may have greater prognostic significance. Regarding subscales, most of the intercorrelations between subscales were moderate: the first factor of AESED relating to the extent to which the caregiver used avoidance and/or modified their routines to accommodate to the patient's symptomatology had the highest association with these dimensions. Although the Turning a Blind Eye subscale had a low association with the rest of the subscales, this subscale nevertheless adequately discriminated clinical variables. This subscale encapsulated 4 items that are more characteristic of bulimia and it is therefore not surprising that carers of people with bulimia scored higher on these items. As our carer sample was comprised primarily of AN families, this might explain why this subscale showed a low association with other subscales.

Different subscales distinguished between the differential thoughts/behaviours resulting from clinical variables. The level of the family accommodation response depended on the type of diagnosis; family members felt more manipulated by patients with anorexia nervosa and families of patients with bulimia nervosa more commonly endorsed an attitude of tolerance of unacceptable behaviours in the Turning a Blind Eye total score (Spearman's rho = 0.35). Impulsive behaviours such as alcohol/illegal substances abuse, threats, self-harm and gambling behaviours were also associated with higher accommodation scores. The level of family accommodation was linked with the hours of face-to-face contact. In contrast to the OCD study, we did not find significant associations with illness severity; it is plausible that in ED it is the comorbid and difficult behaviours that accompany the disorder more than its severity that have a more potent effect on accommodation, carer burden and perhaps outcome. This highlights the need to specifically target these comorbid characteristics in family interventions.

The instrument was sensitive to change after an intervention aimed at changing intrafamilial maintaining factors. Some domains, however, showed much more change than others which suggest that the intervention needs to be modified to target those areas which are resistant to change, such as reassurance seeking (RS) and ignoring disturbing behaviours (TBE). Nevertheless, few caregivers endorsed the turning a blind eye subscale which was more associated with impulsive behaviours and it is therefore possible that the lack of change represents a floor effect or that there was not a large enough sample of carers of people with bulimia nervosa symptomatology. In terms of psychological morbidity as assessed by the HADS, 61.3% of caregivers scored at or above the clinical threshold for anxiety and 20.1% on depression and 56% of carers scored above threshold for high-CC and 65.5% for high-EOI at baseline. The number of caregivers above these clinical threshold points was significantly reduced, following the training. The caregiving burden experienced by the relatives also decreased. The five types of accommodation in eating disorders were related to higher depression and anxiety levels and high expresed emotion in family members. This is similar to what was found in OCD [7].

Our own qualitative findings suggest that many carers who accommodate patients' symptoms do not believe that such accommodation improves the patient's outcome and also report experiencing distress when accommodating to symptoms [39] which corroborates empirical evidence by Calvocoressi and colleagues in OCD [19]. Furthermore, the evidence shows clearly that carers experience psychological morbidity, especially in terms of anxiety at clinical levels, compared to family members of healthy controls, and that over-involved caregivers are particularly adversely affected [3,40].

### Limitations of the Study

There are several limitations to this study. This is a self report measure and families may not have been able to reflect on these processes accurately. Consequently, an expert semi-structured interview might be more reliable and specific. The sample of cases of people with bulimia nervosa was small and so we may not have been able to highlight the specific difficulties that impact on interpersonal relationships in this disorder. Overall, the the factor structure of the results presented in this article may require further scrutiny through a Confirmatory Factor Analysis, particularly to confirm the validity of the total score. It would also be essential to test the validity of the item 28 as a 5-point Likert scale with another sample. Therefore, this item was tentatively retained, pending the results from future data collection. Likewise, the sample may be limited in terms of representativeness due to the high proportion of female carers and the possibility that these carers are more actively involved in seeking help. Thus, a replication of the use of this scale with other carer samples is recommended. In addition, it would have also been of interest to examine whether scores on this instrument were related to more general measures of parenting styles. The intervention was developed before the findings using this instrument were delineated and it is therefore possible that interventions specifically targeted at these interpersonal reactions need to be developed. Finally, family accommodation is thought to fluctuate over time and it is unclear how much of the change is due to repeated measurement or passage of time, therefore longitudinal explorations are needed to identify patient and family factors that may affect changes in accommodation.

## Conclusion

Tailored treatments for the family may be required based on how they organise themselves around the illness and this assessment tool that specifically addresses accommodation and enabling characteristics of each family can be a valuable component in this process. Caregivers can be administered the measure as a self-report questionnaire, possibly as part of the caregivers needs assessment. The AESED is a sensitive instrument for measuring change following family interventions. Different subscales distinguished between the differential thoughts/behaviours associated with diagnosis, co-morbidity, amount of face-to-face contact and whether the carer had had previous eating problems and these had an impact on the profile of accommodation in the family. This measure can therefore be of use for clinical and research contexts and can aid the identification of families that may benefit from interventions targeted at improving family responses and coping strategies.

## Abbreviations

AESED: Accommodation and Enabling Scale for Eating Disorder; FAS: Family Accommodation Questionnaire; OCD: Obsessive Compulsive Disorder; ECI: Experience of Caregiving Inventory; HADS: the Hospital Anxiety and Depression Scale; FQ: Family Questionnaire; EE: Expressed Emotion; CC: criticism; EOI: Emotional Over-Involvement; ECI: Experience Caregiving Inventory; AN: Anorexia Nervosa; BN: Bulimia Nervosa; M: Mean; SD: Standard Deviation; *d*: value for effect size; N: sample size; n.s: no significant.

## Competing interests

The authors declare that they have no competing interests.

## Authors' contributions

ARS conceived and designed the study, oversaw all stages of data collection and performed the statistical analysis and drafted the manuscript. JT coordinated all stages of the study, gave feedback on design and reviewed the manuscript. OK revised the data analysis, interpretation of results and reviewed the manuscript. All authors read and approved the final manuscript.

## Pre-publication history

The pre-publication history for this paper can be accessed here:



## Supplementary Material

Additional file 1 **The 41-original statements for the Accommodation and Enabling Scale for Eating Disorders (AESED)**. The data provided illustrates the 41-items were selected as part of the first questionnaire and the instructions that were given to the caregiver participants. Click here for file
