# Supplementary material for: Development and validation of the Accommodation and Enabling Scale for Eating Disorders (AESED) for caregivers in eating disorders
Source: BMC Health Serv Res. 2009 Sep 23;9:171. doi: 10.1186/1472-6963-9-171 (PMC2759929; doi:10.1186/1472-6963-9-171)
Supplement: Additional file 1 — The 41-original statements for the Accommodation and Enabling Scale for Eating Disorders (AESED). The data provided illustrates the 41-items were selected as part of the first questionnaire and the instructions that were given to the caregiver participants. [file 1472-6963-9-171-S1.doc]

**Appendix A:** **Accommodation and Enabling Scale for Eating Disorders**

Name:…………………………………............Date……………………………………

The following items contain a number of statements that commonly apply to the family members who live with a relatives or friends with an eating disorder. We would like you to read each one and decide how often it has applied to your family members over the **past one month**. It is important to note that there are no right or wrong answers. Your first reaction will usually provide the best answer.

**Items** -During the past month how often have you thought about:

*0 = never*

*1 = rarely*

*2 = sometimes*

*3 = often*

*4 = every day*

| ***Does your relative with eating disorder control:*** PLEASE CIRCLE | | | | | | | | | | |
| --- | --- | --- | --- | --- | --- | --- | --- | --- | --- | --- |
| 1. | the choices of food that you buy? | 0 | | 1 | | 2 | | 3 | | 4 |
| 2. | what other family members do and for how long in the kitchen? | 0 | | 1 | | 2 | | 3 | | 4 |
| 3. | cooking practice and ingredients you use? | 0 | | 1 | | 2 | | 3 | | 4 |
| 4. | what other family members eat? | 0 | | 1 | | 2 | | 3 | | 4 |
| *5. | what other family members do in other rooms in the house? | 0 | | 1 | | 2 | | 3 | | 4 |
| *6. | what other family members can talk about in front of her/him? | 0 | | 1 | | 2 | | 3 | | 4 |
| ***Does your relative engage any family member in repeated conversations:*** | | | | | | | | | | |
| 7. | asking for reassurance about whether she/he will get fat? | 0 | 1 | | 2 | | 3 | | 4 | |
| 8. | about whether it is safe or acceptable to eat certain food? | 0 | 1 | | 2 | | 3 | | 4 | |
| 9. | asking for reassurance about whether she/he looks fat in certain clothes? | 0 | 1 | | 2 | | 3 | | 4 | |
| *10. | about whether or not they would like frequent snacks/meals made for them? | 0 | 1 | | 2 | | 3 | | 4 | |
| *11. | about recipes found in magazines, cookbooks or on TV programmes? | 0 | 1 | | 2 | | 3 | | 4 | |
| 12. | about ingredients and amounts, possible substitutes for ingredients? | 0 | 1 | | 2 | | 3 | | 4 | |
| 13. | about negative thoughts and feelings | 0 | 1 | | 2 | | 3 | | 4 | |
| 14. | about self-harm | 0 | 1 | | 2 | | 3 | | 4 | |
| ***Do any family members have to accommodate to the following:*** | | | | | | | | | | |
| 15. | what crockery is used? | 0 | 1 | | 2 | | 3 | | 4 | |
| 16. | how crockery is cleaned? | 0 | 1 | | 2 | | 3 | | 4 | |
| 17. | what time food is eaten? | 0 | 1 | | 2 | | 3 | | 4 | |
| 18. | what place food is eaten? | 0 | 1 | | 2 | | 3 | | 4 | |
| 19. | how the kitchen is cleaned? | 0 | 1 | | 2 | | 3 | | 4 | |
| 20. | how food is stored? | 0 | 1 | | 2 | | 3 | | 4 | |
| 21. | the exercise routine of the relative with an ED? | 0 | 1 | | 2 | | 3 | | 4 | |
| 22. | your relative’s checking their body shape or weight? | 0 | 1 | | 2 | | 3 | | 4 | |
| 23. | how the house is cleaned and tidied? | 0 | 1 | | 2 | | 3 | | 4 | |
| ***Do you choose to ignore aspects of your relative’s eating disorder that impinge your family’s life in an effort to reconcile or make it tolerable for the rest of the family such as if:*** | | | | | | | | | | |
| 24. | food dissapears? | 0 | 1 | | 2 | | 3 | | 4 | |
| 25. | money is taken? | 0 | 1 | | 2 | | 3 | | 4 | |
| 26. | the kitchen is left a mess? | 0 | 1 | | 2 | | 3 | | 4 | |
| 27. | the bathroom is left a mess? | 0 | 1 | | 2 | | 3 | | 4 | |
| 28. In general, to what extent would you say that the relative with an eating disorders controls family life and activities?  *None at All About Half Completely*  0 1 2 3 4 5 6 7 8 9 10 | | | | | | | | | | |
| **To continue answering the questionnaire, please bear in mind the following:**  If it has never happened you would CIRCLE the number 0, if it has happened 1-3 times per month you would CIRCLE the number 1. If it has happened 1-2 times per week, then you would CIRCLE the number 2. If it has happened 3-6 times per week you would CIRCLE the number 3, and if happens daily you would CIRCLE the number 4. Over the **past one month**.  *0 = never*  *1 = 1-3 times/month*  *2 = 1-2 times/week*  *3 = 3-6 times/week*  *4 = daily* | | | | | | | | | | |
| *29. | How often did you reassure your relative during the past month? | 0 | 1 | | 2 | | 3 | | 4 | |
| *30. | How often did you provide items for the patient’s compulsions? | 0 | 1 | | 2 | | 3 | | 4 | |
| 31. | How often did you participate in behaviours related to your relative’s compulsions? | 0 | 1 | | 2 | | 3 | | 4 | |
| 32. | How often did you assist your relative in avoiding things that might make her/him more anxious? | 0 | 1 | | 2 | | 3 | | 4 | |
| If it the answer is NO you would CIRCLE the number 0, if the answer is MILD you would CIRCLE the number 1. If the answer is MODERATE, then you would CIRCLE the number 2. If the answer is SEVERE you would CIRCLE the number 3, and if the answer is EXTREME you would CIRCLE the number 4. Over the **past one month**.  *0 = no*  *1 = mild*  *2 = moderate*  *3 = severe*  *4 = extreme* | | | | | | | | | | |
| 33. | Have you avoided doing things, going places, or being with  people because of your relative’s disorder? | 0 | 1 | | 2 | | 3 | | 4 | |
| 34. | Have you modified your family routine because of your relative’s symptoms? | 0 | 1 | | 2 | | 3 | | 4 | |
| *35. | Have you had to do some things for the family that are usually  your relative’s responsibility? | 0 | 1 | | 2 | | 3 | | 4 | |
| 36. | Have you modified your work schedule because of your relative’s needs? | 0 | 1 | | 2 | | 3 | | 4 | |
| 37. | Have you modified your leisure activities because of your relative’s needs? | 0 | 1 | | 2 | | 3 | | 4 | |
| 38. | Has helping your relative in the before mentioned ways caused you distress? | 0 | 1 | | 2 | | 3 | | 4 | |
| 39. | Has your relative with an eating disorder become distressed/anxious when you have not provided assistance? If YES, to what degree?we cannot ask this | 0 | 1 | | 2 | | 3 | | 4 | |
| 40. | Has your relative become angry/abusive when you have not  provided assistance? | 0 | 1 | | 2 | | 3 | | 4 | |
| *41. | Has your relative with an ED spent more time completing rituals when you have not provided assistance? | 0 | 1 | | 2 | | 3 | | 4 | |

***Deleted items**
